# Kinetic effects yield different results on the timescales of laboratory and synchrotron high-pressure experiments

**DOI:** 10.1107/S2052252526001569

**Published:** 2026-02-24

**Authors:** Julien Haines

**Affiliations:** a Institut Charles Gerhardt Montpellier Université de Montpellier, CNRS, ENSCM 34293Montpellier France

**Keywords:** high pressure, polymorphism, incommensurate structures, laboratory X-ray diffraction, synchrotron radiation, pharmaceuticals

## Abstract

Kinetic effects can be a critical factor in the study of high-pressure phase transitions. X-ray diffraction experiments on the timescales of the laboratory and the synchrotron can provide complementary results on such transformations.

High pressure is a powerful tool for probing the structure of crystalline solids (Tse, 2020 ▸; Boldyreva, 2010 ▸). Pressure as a thermodynamic variable allows the volume of a crystal to be varied with minor impact on thermal motion, modifying interatomic potentials and crystal structures. High-pressure studies can be readily implemented on both laboratory sources and dedicated beamlines at synchrotron sources, either with compact diamond anvil cells or large-volume presses.

High pressure is particularly useful for studying of the stability of polymorphs of pharmaceutically active compounds, as the manufacturing, processing and administration of drugs depend on the properties of the polymorph present (Boldyreva, 2010 ▸). The paper by Bogdanov *et al.* (2026 ▸) in the current issue of *IUCrJ* presents a detailed investigation of the high-pressure phase transitions in δ-chlorpropamide. Chlorpropamide [1-(4-chlorophenyl)­sulfonyl-3-propylurea], which belongs to the sulfonyl­urea class of compounds, is a drug used to treat type-2 diabetes. This compound exhibits a very high number of polymorphs – at least twelve under various pressure and temperature conditions. The polymorphism arises from the high number of possible conformations of the aromatic ring and the alkyl terminal group. The delta form investigated under pressure in the work described by Bogdanov *et al.* is the densest known polymorph of chlorpropamide. Their single-crystal X-ray diffraction investigation under high pressure is particularly rigorous, in that four experiments were performed using different protocols and experimental setups on laboratory and synchrotron sources. Experiments were performed on two laboratory diffractometers, an Oxford Diffraction Gemini R Ultra and a Rigaku Synergy-S Dualflex, and two different beamlines at the European Synchrotron Radiation Facility, BM01 (Dyadkin *et al.*, 2016 ▸) and ID27 (Mezouar *et al.*, 2024 ▸).

These various experimental setups are characterized by their very different, characteristic, acquisition times due to orders of magnitude differences in brilliance. Additionally, the X-ray wavelengths differ by a factor of two, which affects the maximum resolution. At higher energies and fluxes, radiation damage may also occur. These factors will influence data quality, particularly in terms of precision of lattice parameters and atomic positions. Furthermore, the results will depend on the kinetics of any high-pressure phase transitions.

Based on these single-crystal X-ray diffraction measurements, a new commensurate high-pressure phase with a tripled *b* cell parameter was identified. The results were found to vary significantly depending on the instrument used because of their intrinsic characteristics. Using the oldest laboratory diffractometer, structure refinements were not possible and difficulties were encountered even in obtaining precise lattice parameters. In contrast, the phase transition was clearly evident using data acquired with the more recent Rigaku Synergy-S Dualflex diffractometer, which was equipped with a microsource.

Very different results were obtained using synchrotron radiation, with the observation of an incommensurate phase featuring a sequence of molecular conformations along the modulation vector, followed by a lock-in transition to the commensurate phase at much higher pressure. While full structure determination of the high-pressure phase was possible with the BM01 data, experiments on the high-flux ID27 beamline were hindered by radiation damage. It was necessary to attenuate and defocus the beam and only the cell parameters could be measured. In spite of these constraints, less time was required for data acquisition and the lock-in transition was observed at even higher pressure than on BM01. The structure remained commensurate on pressure release before reverting directly to the low-pressure form (see Fig. 1 ▸).

These results show the need to carefully adapt experimental protocols to the nature of the system being studied, in terms of sensitivity to radiation damage and to kinetic effects. Bogdanov *et al.* concluded that kinetic factors play a critical role in the high-pressure phase transitions in chlorpropamide. Notably, the pressure required to obtain the high-pressure commensurate phase decreased significantly with increasing acquisition time. The most extreme case was observed during the prolonged laboratory measurements, where the transition pressure was much lower and no intermediate incommensurate form was observed. Such long experiments would be poorly suited for synchrotron sources. Future work on molecular crystals with multiple polymorphs could include measurements at synchrotron sources of the effects of time and temperature on the high-pressure phase transitions.

In summary, the work of Bogdanov *et al.* can be considered as a case study providing valuable insight concerning kinetic effects on phase transitions at high pressure and the need to critically evaluate the experimental protocols used in different studies. A key take-home message from their study is the complementarity of laboratory and synchrotron data. 

## Figures and Tables

**Figure 1:**
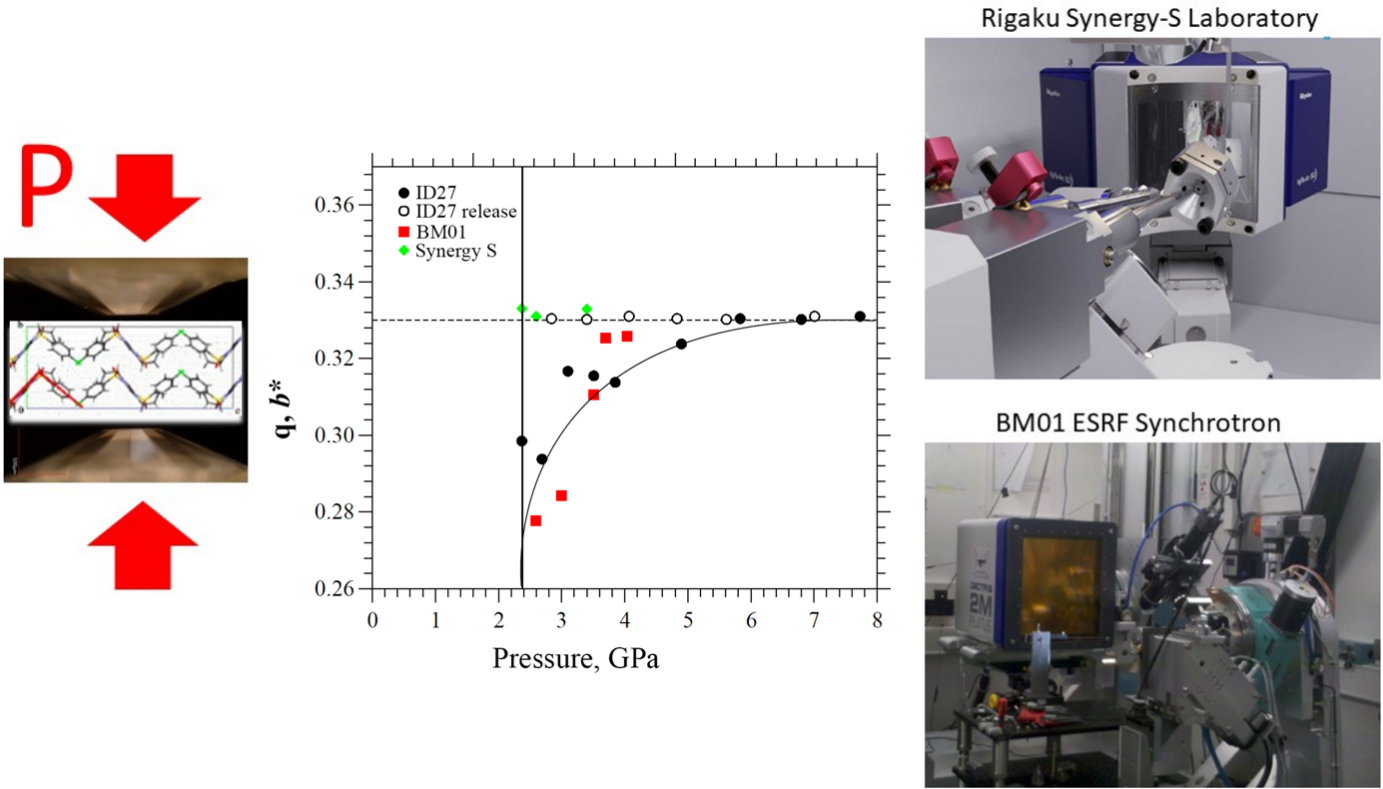
Upon compression of δ-chlorpropamide, direct transformation to a commensurate (**q** = 0.33) high-pressure phase is observed in laboratory single-crystal X-ray diffraction experiments, whereas an intermediate incommensurate (**q** < 0.33) phase is observed in synchrotron experiments owing to kinetic effects. [The structure and the pressure plot are reproduced from Bogdanov *et al.* (2026).]

## References

[bb5] Bogdanov, N. E., Rashchenko, S. V., Zakharov, B. A., Seryotkin, Y. V. & Boldyreva, E. V. (2026). *IUCrJ* **13**, 146–158. 10.1107/S2052252525011601 PMC12951832 41557337

[bb1] Boldyreva, E. (2010). *High-Pressure Crystallography: From Fundamental Phenomena to Technological Applications*, edited by E. Boldyreva & P. Dera, pp. 533–543. Springer Science+Business Media B. V.

[bb2] Dyadkin, V., Pattison, P., Dmitriev, V. & Chernyshov, D. (2016). *J. Synchrotron Rad.* **23**, 825–829. 10.1107/S1600577516002411 27140164

[bb3] Mezouar, M., Garbarino, G., Bauchau, S., Morgenroth, W., Martel, K., Petitdemange, S., Got, P., Clavel, C., Moyne, A., Van Der Kleij, H., Pakhomova, A., Wehinger, B., Gerin, M., Poreba, T., Canet, L., Rosa, A., Forestier, A., Weck, G., Datchi, F., Wilke, M., Jahn, S., Andrault, D., Libon, L., Pennacchioni, L., Kovalskii, G., Herrmann, M., Laniel, D. & Bureau, H. (2024). *High Pressure Res.* **44**, 171–198.

[bb4] Tse, J. S. (2020). *Natl Sci. Rev.* **7**, 149–169. 10.1093/nsr/nwz144 PMC8289026 34692029

